# Parasitoid Distribution and Parasitism of the Fall Armyworm *Spodoptera frugiperda* (Lepidoptera: Noctuidae) in Different Maize Producing Regions of Uganda

**DOI:** 10.3390/insects12020121

**Published:** 2021-01-29

**Authors:** Michael Hilary Otim, Stella Adumo Aropet, Moses Opio, Dalton Kanyesigye, Henry Nakelet Opolot, Wee Tek Tay

**Affiliations:** 1 National Crops Resources Research Institute (NaCRRI), Kampala P.O. Box 7084, Uganda; stellaadumo@yahoo.com (S.A.A.); opio.moses@yahoo.com (M.O.); kanyesigyedalton@gmail.com (D.K.); 2 Ministry of Agriculture, Animal Industry and Fisheries, Entebbe P.O. Box 102, Uganda; hnopolot@gmail.com; 3 Black Mountain Laboratories, Clunies Ross Street, Commonwealth Scientific and Industrial Research Organisation, Canberra 2601, Australia; Weetek.Tay@csiro.au

**Keywords:** *Charops* cf. *diversipes*, *Chelonus bifoveolatus*, *Coccygidium luteum*, *Cotesia flavipes*, *Cotesia icipe*, *Sturmiopsis parasitica*, *Telenomus remus*, beneficial insects

## Abstract

**Simple Summary:**

The fall armyworm (FAW), a native of the Americas that was confirmed in Africa in 2016, has been reported to cause substantial damage to maize and sorghum in all sub-Saharan African countries. In Uganda, farmers rely mainly on synthetic insecticides, which can be harmful to humans, the environment, and significantly increase the cost of production. To lessen the disadvantages associated with synthetic insecticides, the use of parasitoids could be exploited. Fall armyworm parasitoids have been reported from the Americas, Asia, and some African countries, but not from Uganda. In this study, we aimed to determine the identity and distribution of FAW parasitoids in Uganda. We found 13 species of parasitoids attacking FAW in the surveyed locations. These included 11 species of insects in the wasp order and two in the fly order. Four of these are wasps that attack the eggs of FAW, while the remaining seven wasps and two fly species attack the larvae of FAW. Two wasp genera (*Chelonus* and *Coccygidium* spp.) were more abundant and widely distributed when compared with the other parasitoid species. All parasitoids contributed to an average of 9.2% FAW larval mortality rate across the study locations.

**Abstract:**

The fall armyworm (FAW) *Spodoptera frugiperda* (J.E. Smith) (Lepidoptera: Noctuidae) has successfully invaded Africa, where it has significantly impacted maize and sorghum production. Management of FAW in Africa predominantly relies on synthetic insecticides, which are expensive, and negatively impact the environment and beneficial insects. We, therefore, conducted field surveys in Uganda in 2017 and 2019 to identify egg and larval parasitoids of FAW for possible use in integrated pest management (IPM) programs. Parasitoids were identified by their mitochondrial DNA cytochrome c oxidase subunit 1 (mtCOI) gene sequences. We identified 13 parasitoid species belonging to three families of Hymenoptera: Platygastridae, Braconidae and Ichneumonidae, as well as one Dipteran family (Tachinidae). *Coccygidium* spp. and *Chelonus bifoveolatus* were the most abundant and widely distributed parasitoids. Overall, parasitism averaged 9.2% and ranged from 3.1% to 50% in 2017, and 0.8% to 33% in 2019. Parasitism by *Sturmiopsis parasitica*, *Diolcogaster* sp., and *Cotesia flavipes* on FAW in maize crops are reported for the first time. Our results suggest high biological diversity of FAW parasitoids, which should be exploited in the IPM of the FAW in Uganda.

## 1. Introduction

The fall armyworm (FAW) *Spodoptera frugiperda* (J.E. Smith) (Lepidoptera: Noctuidae), a native to the tropical and sub-tropical regions of the Americas, was first reported in West Africa from Nigeria, and the island of São Tomé and Principe in 2016 [1], and subsequently in almost all sub-Saharan African countries [2,3,4,5,6,7]. The estimated yield losses ranged from 8.3 to 20.6 million tons of maize in Africa, valued yearly at USD 2481–6187 million [8]. In Uganda, FAW was estimated as capable of causing maize losses of 558.9 to 1391 tons annually under no control, translating to USD 163.7 to 407.5 million annually [8]. However, recent field studies in Uganda found that FAW, which is now in all maize growing districts of Uganda, can cause grain yield losses of up to 50% or more under severe infestation and drought conditions (MH Otim, unpubl. data), highlighting the economic impact of this invasive pest in East Africa.

In the Americas, chemical insecticides, genetically modified maize/cotton/soy, resistant varieties, and augmentative control methods are used to manage FAW [8]. In Africa, smallholder farmers manage FAW using synthetic insecticides [9,10,11], and several other practices including early planting, handpicking of FAW eggs and larvae, frequent weeding, manure and fertilizer applications, destruction of infested plants, application of sand and ash, crop rotation, habitat management (e.g., maize intercropped with Greenleaf desmodium (*Desmodium intortum*)), with *Bracharia* planted around the intercrop [12], and intercropping. Cultural practices alone, however, have limited effectiveness against this pest given its high migratory behavior and reproductive capacity [13]. The use of insecticides is associated with increased costs of production, dangers to human health, and negative impact to the environment and beneficial insects [14]. Prolonged use of synthetic insecticides could lead to the development of resistance in target pest species. Insecticide resistance in FAW was detected in the New World (e.g., [15,16,17]) as well as in introduced populations (e.g., [18,19,20]) and this generates economic and biosecurity concerns. The management of FAW should, therefore, explore alternative options including the use of beneficial insects where possible.

Integrating biological control for more sustainable control of the pest has been encouraged in many African countries, including Uganda [21]. The use of introduced or locally occurring biological control agents can reduce FAW outbreaks and provide a more economic and environmentally friendly option. Natural enemies are an important mortality factor of FAW in several places. The reported natural enemies include fungi [22,23], bacteria [24], viruses and Microsporidia [24], parasitoids [21], and entomopathogenic nematodes [23]. In the Americas, FAW is attacked by an assemblage of parasitoids (approximately 150 species) [22,23,25,26,27,28,29,30,31,32,33]. Across the invasive range of FAW, several parasitoids have been identified (e.g., China [34]; India [35,36]). In Africa, reports of parasitoids attacking FAW have come from South Africa, Côte d’Ivoire, Ethiopia, Kenya, Niger, Benin, and Tanzania [37,38,39]. The lack of studies on parasitoids attacking FAW from Uganda, therefore, limits the development of biological control of this pest in the country.

Here, we report on an exploratory study of the occurrence of FAW parasitoids, their relative abundance, and parasitism in maize crops from eastern, central, northern, and western regions of Uganda in 2017 and 2019. We used a combination of morphological and molecular species diagnostic methods to identify the parasitoid species. We highlight existing challenges and outline future research opportunities on potential uses of these FAW parasitoids as candidate biological control agents for integrated pest management (IPM) of this important pest.

## 2. Materials and Methods

### 2.1. Survey for FAW and Natural Enemies

**Figure 1:**
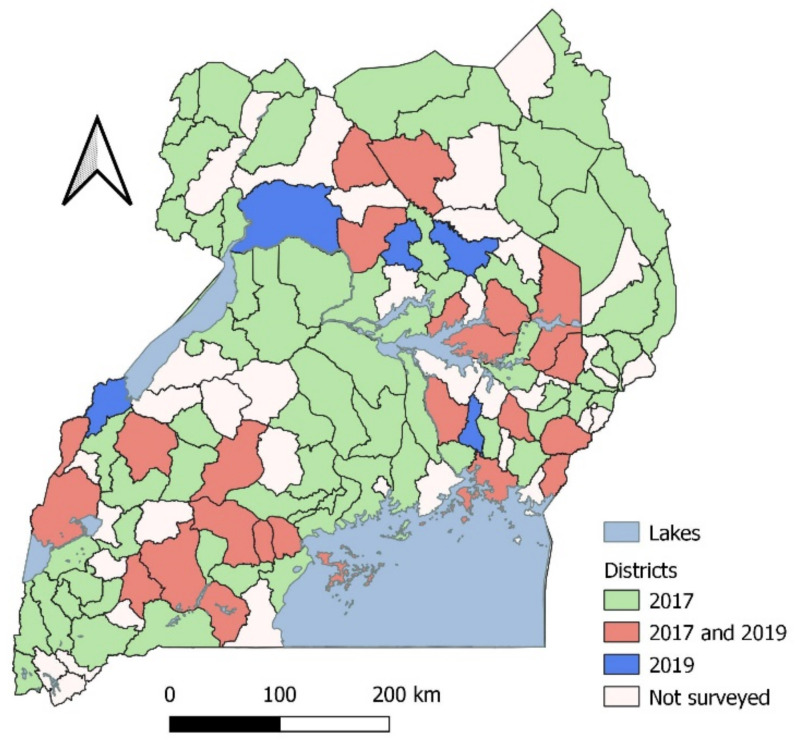
Map of Uganda showing districts in which surveys for fall armyworm parasitoids were conducted in 2017 (green), 2019 (dark blue), and 2017 and 2019 (red). Districts not surveyed have been left blank.

We conducted surveys for FAW parasitoids in 99 districts of Uganda across the eastern, central, northern, and western regions (Figure 1) in 2017 and 2019. In each region, representative maize producing districts were surveyed based on guidance by the District Production Departments and availability of maize in the field. We sampled 50 maize plants per field from two or three sub-counties (two fields per sub-county) per district, separated by at least 3 km. While walking diagonally across the field, each selected plant was visually inspected for the presence of FAW eggs and larvae. The eggs and larvae of FAW were identified based on the described characteristics [31]. Egg batches were placed in petri dishes containing a piece of moistened filter paper while larvae were individually placed in propylene vials, and provided with maize leaf as food. The collected samples were transported to the laboratory for further observation.

### 2.2. Rearing and Identification of Parasitoids

Collected FAW eggs and larvae were incubated at 12:12 hr light:dark duration, RH 65.5 ± 0.27%, and temperature 25.8 ± 0.071 °C in the laboratory for emergence of parasitoids, or FAW larvae and adults, respectively. For adult parasitoid emergence or pupa pupal development, we kept the larvae on fresh maize leaves, which were replenished as needed. Emerged parasitoids were recorded daily and preserved in 90% ethanol. We did not dissect the dead larvae and pupae to check for the presence of parasitoids. Emerged parasitoids were examined and identified to the family/genus level using published identification guides for Platygastridae [40], Braconids [41,42,43], Ichneumonidae [44], and Tachinidae [45,46]. This was followed by DNA barcoding to confirm the identity of the parasitoids (details described below). Voucher specimens are stored at the National Crops Resources Research Institute, Namulonge, Uganda. The samples used for DNA barcoding comprised 40 individuals (Coccygidium spp (9), Chelonus sp (9), Tachinidae (6), Telenomus (4), unknown Platygastridae (1), Diolcogaster (1), Meteorus (1), Cotesia spp (4), and Charop sp (5)). The number was dictated by the number of individuals of each taxon that were reared.

### 2.3. DNA Extraction

The DNA from individual parasitoids was extracted using the chelex method [5]. All insects were rinsed twice with sterile molecular grade water (Thermo Fisher Scientific, UK) to wash off the ethanol used for preservation. Using a clean sterile surgical blade, a leg of each specimen was excised and placed in a sterile 1.5 mL micro-centrifuge tube. For smaller parasitoids (e.g., *Telenomus* spp. and *Cotesia* spp.) the entire insect was used. For each specimen, we added 50 μL of 10% Chelex 100 solution followed by 10 μL (20 mg/mL) of proteinase K solution. Each sample was incubated at 56 °C overnight, followed by a brief vortex then heat inactivation at 100 °C for 15 min. The resulting mixture was centrifuged at 15,900 relative centrifugal forces (rcf) for 3 min and 40 μL supernatant was stored at −20 °C. This stock DNA was used for PCR amplification. The unused portion of parasitoids was stored in absolute ethanol and kept at −20 °C for future reference.

### 2.4. PCR Amplification of COI Segments

The mitochondrial DNA cytochrome c oxidase subunit 1 (mtCOI) gene of all parasitoid samples was amplified by polymerase chain reaction (PCR) using the LCO1490 and HCO2198 primer set [47] in a 25 µL volume per reaction. The PCR mixture consisted of 16.25 µL of nuclease free water (ThermoFisher Scientific), 2.5 µL of 10× Dream Taq green buffer, 0.5 µL deoxynucleotide triphosphate (dNPTs) (10 mM), 1 µL of each primer (10 Pmol/µL), 0.25 µL of Dream Taq DNA polymerase (ThermoFisher-Scientific), 2.5 µL of 5% Tween 20 and 1 µL of the template DNA. The PCR thermo-cycling conditions were: 94 °C (2 min) for initial denaturation, followed by 35 cycles of 94 °C (30 s), 52 °C (35 s), and 72 °C (45 s), and a final extension step at 72 °C for 10 min. Post PCR reactions were stored at 4 °C until needed. PCR products were visualized by electrophoresis in 1× TAE buffer 1.5% (*w*/*v*) agarose gels (UltraPure Agarose, Invitrogen, Carlsbad, CA, USA) stained with 5 µL of ethidium bromide, visualized under UV light to confirm the presence of amplicon products, and photographed using a digital camera in U: GENIUS3 gel documentation system.

### 2.5. Sequencing and Sequence Analysis

All PCR products were sent to Macrogen Europe (1105 AZ, Amsterdam, the Netherlands) for purification and sequencing. We used the Pregap4 and Gap4 sequence analysis programs within the Staden sequence analysis package [48] to analyze trace files and assemble contigs. Nine sequences (Coccygidium (5) and Chelonus (4)) were discarded either because they were short or dirty sequences. Geneious^®^ 11.1.3 (https://www.geneious.com) [49] was used to translate DNA nucleotide sequences to protein sequences using invertebrate mitochondrial genetic code, and choosing the appropriate reading frame to confirm the absence of premature stop codons. All our partial sequences were not pseudogenes or nuclear mitochondrial (NuMT) sequences because of the absence of premature stop codons. Assembled partial mtCOI sequences from all parasitoid specimens were compared to sequences in GenBank via Blast search program against the non-redundant (nr) DNA database [50], and where necessary compared also to the International Barcode of Life (iBoL) database [51] to assist with species molecular identification.

### 2.6. Phylogenetic Analysis

Phylogenetic analyses were not carried out where there was limited information available from GenBank or iBoL for assignment of parasitoid identity (i.e., only to Family level, e.g., Platygastridae), or where species identity could be confidently assigned due to a high degree of shared nucleotide identity. Phylogenetic analyses were performed if our unknown parasitoid sequences shared a high degree of nucleotide similarity known from iBoL or GenBank. Phylogenetic analyses were carried out for the two hymenopteran genera as described below, without rooting because our interest was not to define their phylogenies but rather to ascertain if our sequences would cluster with particular taxa and their associated statistical confidence. 

#### 2.6.1. *Telenomus* spp.

Two of the four *Telenomus* mtCOI sequences (MT780201, MT780202) shared a high level of nucleotide similarity with *T. remus*. To determine the species status of the remaining *Telenomus* samples (MT782153, MT782154), we applied a phylogenetic approach by combining these unknown sequences with other *Telenomus* genus/Platygastridae family sequences from GenBank (accessed 12 July 2020). Sequences downloaded from GenBank and our five Platygastridae candidate sequences were imported into Geneious 11.1.5 (Biomatters Ltd., Auckland, New Zealand) for alignment using MAFFT alignment v7.450 [52,53] with default settings (Algorithm: Auto; Scoring Matrix: 200 PAM/K = 2; Gap open penalty: 1.53; Offset value: 0.123). All sequences were trimmed to 560 bp to retain maximum sequence length. Sequences shorter than 560 bp and excess identical (redundant) sequences sharing 100% sequence identity were excluded while retaining a maximum of two identical sequences for each unique haplotype. The resulting non-redundant sequences were analyzed using IQ-Tree [54] <http://iqtree.cibiv.univie.ac.at> with the automatic substitution model and by selecting 1000 ultrafast bootstrap replication options to estimate the confidence of branch nodes. The resulting unrooted phylogeny was visualized and presented using Dendroscope (version 3.5.7, built 30 Jan 2016; [55]) (Figure S1).

#### 2.6.2. *Coccygidium* spp.

A total of 44 partial mtCOI sequences from the iBoL (*n* = 33) and GenBank (*n* = 11) were downloaded (accessed 26 April 2020), as well as two outgroup individuals (*Agathis* sp. H15103 (KP943649); *Agathis* sp. H15112 (KP943651); GenBank accessed 12 December 2020), and aligned with our three Ugandan *Coccygidium* sequences from GenBank (MT784160, MT784186, MT784187). We removed redundant sequences (i.e., those sharing 100% sequence identity) and short sequences, and trimmed the remaining 596 bp for maximum sequence length. Trimmed sequences were aligned and used to infer phylogenetic relationships to help assess the confidence of species assignment based on nucleotide similarity. Inference of the *Coccygidium* spp. phylogeny and phylogenetic tree visualization with the *Agathis* individuals as the outgroup were as described for *Telenomus* spp.

### 2.7. Relative Abundance and Parasitism Rates

Relative abundance (RA) was expressed as a percentage of the total number of parasitoids of a particular species (ni) to the total number of all parasitoids that were reared (N) (Equation (1)).

(1) $$RA=\left(ni÷N\right)\times 100$$

Percent of parasitism (Para) by different species was expressed as the ratio of the total number of parasitized FAW larvae (*n*) and the total number of FAW larvae collected from a particular district (N). The figures were adjusted for the gregarious larval parasitoid (*C. flavipes*) that had more than one adult parasitoid emerging from a single larva. In this case, the number of parasitized larvae was taken as one regardless of the number of parasitoids that emerged from it.

## 3. Results

In 2017, we recovered parasitoids from 32 of the 96 districts sampled. In 2019, we recovered parasitoids from 18 of the 31 districts sampled.

In total, we recovered 13 species attacking FAW in the surveyed locations, including egg, egg/larval, larval, and larval/pupal parasitoids (Table 1). The egg parasitoids included two species of *Telenomus*: *T. remus* Nixon and *Telenomus* sp., and an unidentified Platygastrid (all Hymenoptera: Platygastridae) that matched to *Telenomus sp.* at 87.44% nucleotide identity. The sequences of the five egg/larval parasitoids recovered from FAW matched *Chelonus bifoveolatus* Szépligeti (Hymenoptera: Braconidae) with a 99.8% to 100% sequence identity. The larval parasitoids included *Coccygidium luteum* (Brullé, 1846), *Coccygidium* sp., *Cotesia flavipes* (Cameron), *Cotesia icipe* Fernandez-Triana and Fiaboe, and *Diolcogaster* sp. (all Hymenoptera: Braconidae) and *Charops* cf. *diversipes* (Hymenoptera: Ichneumonidae). The larval/pupal parasitoids were *Meteorus* sp. (Hymenoptera: Braconidae) and two species of Tachinidae: *Sturmiopsis parasitica* (Curran) and *Drino quadrizonula* (Thomson).

### 3.1. Distribution of Egg Parasitoids

***Telenomus remus:*** This constituted 60% of the egg parasitoids reared, and was recovered from FAW eggs collected from Bukedea and Ntoroko Districts, eastern and western regions, respectively (Table 2).

***Telenomus* sp.** constituted 37% of the total egg parasitoids reared and was 96.6% identical to *T. remus* (Table 1). This *Telenomus* sp. was recovered from Soroti District (eastern region) and Mbarara (western region).

***Platygastridae* sp.** This taxon shared 87.3% identity with *Telenomus* sp. (GenBank MK533751) (Table 1), and accounted for 3% of the egg parasitoids (Table 2). It was recovered from Ntoroko district in Western Uganda.

**Phylogenetic relationships among the Platygastridae**

We used a total of 193 Platygastroidea and one Chalcidoidea partial mtCOI sequences that included species within the *Telenomus* genus (from 250 GenBank downloaded sequences; accessed 12 July 2020) for phylogenetic inferences (and also included our four detected candidate *Telenomus* sequences and one Platygastridae sequence) to help assess their species status. Uncorrected pairwise nucleotide distances (*p*-dist) between all 199 trimmed and aligned sequences ranged from 0% to 17.9% (i.e., 82.1–100% sequence identity). We identified at least 21 subclades within the trimmed and aligned sequences, with at least 13 subclades containing sequences from specimens previously identified as *Telenomus* species (Figure S1).

Based on the uncorrected *p*-dist estimates, the Ugandan *Telenomus* sequences (GenBank MT780201, MT780202) shared 100% sequence identity with *T. remus* (MK533751) and clustered confidently (99% node support value) with the partial mtCOI sequences of five previously reported *T. remus* individuals from Benin (MK533751), India (KT305960, KP994550), and China (MN123243, MN123244) (*p*-dist: 0–0.53%). Two other sequences (GenBank MT780153, MT780154) clustered as a basal sister species with *T. remus* (*p*-dist: 4.07–7.26%) and *Telenomus* spp. from Canada (*p*-dist: 8.14–8.85%), suggesting that the two identical *Telenomus* sequences (GenBank MT780153, MT780154) from Uganda likely represented a novel African species that was also capable of parasitizing FAW.

The Ugandan Platygastridae sequence (GenBank accession no. MT784162) was the most divergent sequence amongst the 194 sequences from the Platygastroidea/Chalcidoidea Superfamilies with nucleotide similarity that ranged from 82.14% (MG355758, *Platygastridae* sp.)/ 86.25% (MH926817, Chalcidoidea) to 87.86% (KR803447, *Telenomus* sp.).

### 3.2. Distribution of Egg/Larval Parasitoids of Spodoptera Frugiperda

#### *Chelonus bifoveolatus* 

We reared *Che. bifoveolatus* from larvae of FAW in both 2017 and 2019 (Table 3). Our sequences shared 99.6% to 100% identity with *Che. bifoveolatus* reported in Ghana, Benin, and French Polynesia (GenBank KX052941, MN900743, MN900744).

In 2017, *Chelonus bifoveolatus* was recovered from 14 districts (Alebtong, Dokolo, Kaabong, Kaberamaido, Kasese, Katakwi, Kole, Kotido, Kumi, Mayuge, Nwoya, Pakwach, Serere and Soroti districts; Table 3). In 2019, *Che. bifoveolatus* was recovered from nine districts (Alebtong, Kasese, Katakwi, Kole, Luuka, Mayuge, Nwoya, Serere, and Soroti) in eastern, northern, and western regions (Table 4).

### 3.3. Distribution of Larval Parasitoids of Spodoptera Frugiperda

The larval parasitoids recovered included two species of *Coccygidium* (*Coc. luteum* and *Coccygidium* sp.), two species of *Cotesia* (*Cot. flavipes* and *Cot. icipe*), and a *Diolcogaster* sp. (Table 1).

#### 3.3.1. *Coccygidium Luteum* and *Coccygidium* sp.

**Figure 2:**
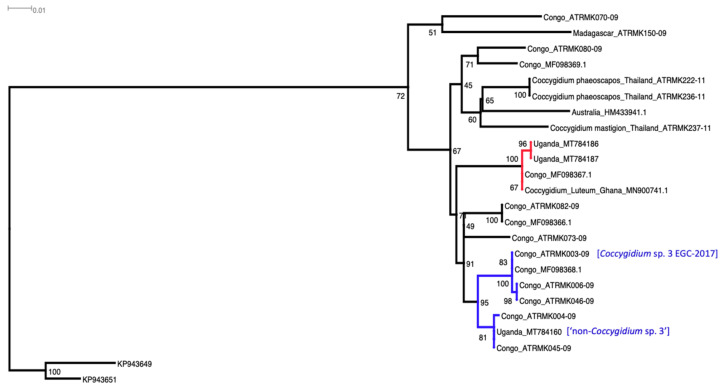
Phylogenetic placements of the Ugandan *Coccygidium* species, with two of the three sequences (GenBank MT784186, MT784187) being shown to cluster confidently with *Coc. luteum* from Ghana (MN900741) and Congo (MF098367), and the remaining one (GenBank MT784160) clustered with sequences as the sister clade to the “*Coccygidium*. sp. 3 EGC-2017” individual (GenBank MF098368). The outgroup individuals were *Agathis* spp. from Mexico (KP943649) and the United States of America (KP943651) [59].

Our *Coccygidium luteum* (MT784186, MT784187) shared 99.7% sequence identity with the *Coc. luteum* reported from Ghana (GenBank accession MN900741) and the Republic of Congo (MF098367; iBoL: ATRMK048-09), and clustered confidently (100%) as a clade (Figure 2, red branches). It was recovered from five districts in Eastern and Northern Uganda in the 2017 survey (Table 3) and two districts in the 2019 survey in Eastern Uganda (Table 4). The remaining one *Coccygidium* sequence from Uganda (MT784160) clustered with a high node support value of 81% with two *Coccygidium* sequences from the Republic of Congo (i.e., iBOL accession numbers ATRMK004-09, ATRMK045-09). This formed a sister-clade (95% node confidence value) with four *Coccygidium* sequences (i.e., iBoL accession numbers ATRMK003-09, ATRMK006-09, ATRMK046-09; GenBank MF098368, named as “*Coccygidium* sp. 3 EGC-2017” [58]), also from the Republic of Congo, and these sequences (blue branches) appeared paraphyletic as sister clades to *Coc. luteum* (with an average uncorrected *p*-dist of 3.8%, data not shown), suggesting that at least another undescribed “non-*Coccygidium* sp. 3” was present in both Uganda and the Republic of Congo and could use FAW as a reproductive host. This unidentified Ugandan *Coccygidium* sp. (MT784160) was recovered from 15 districts in 2017 survey in eastern and northern regions (Table 3). In the 2019 survey, we only recovered the “non-*Coccygidium* sp. 3” (GenBank MT784160) from five districts in central, northern, and western regions (Table 4). 

#### 3.3.2. *Cotesia flavipes*

The *Cotesia flavipes* recovered in this study has high sequence homology matching with previously reported *Cot. flavipes* partial mtCOI sequences (i.e., 100% matched GenBank JF865973 and 99.6% matched GenBank MN900741). *Cot. flavipes* was recovered only in the 2019 survey from Nwoya District in Northern Uganda (Table 4).

#### 3.3.3. *Cotesia Icipe*

The *Cotesia icipe* that we recovered in this study had a partial mtCOI gene sequence that matched 100% with *Cot. icipe* (BOLD museum ID JMIC0355) and 99.6% matched with MN900741 reported in Ghana (Table 1). This parasitoid was recovered only in the 2019 survey from Busia and Kamuli Districts in Eastern Uganda, and Kasese District in Western Uganda (Table 4).

#### 3.3.4. *Diolcogaster* sp.

This species was recovered from Oyam in Northern Uganda in 2019 (Table 4) and shared 98.38% nucleotide sequence identity with a previously reported *Diolcogaster* sp. from Guanacaste, Costa Rica (GenBank HM397609), suggesting that these two *Diolcogaster* specimens were potentially the same or very closely related species.

### 3.4. Distribution of Larval/Pupal Parasitoids of Spodoptera Frugiperda

We recovered four species of larval/pupal parasitoids. These were two hymenopteran species (*Meteorus* sp., *Charops* cf**.** *diversipes*), and two dipteran species (*Sturmiopsis parasitica* and *Drino quadrizonula*).

#### 3.4.1. *Meteorus* sp.

The *Meterous* species shared 99.84% of nucleotide with the *Meteorus* sp. recovered from Senegal (GenBank MF673599, Table 1) and was, therefore, likely to be the same species. This parasitoid was recovered from FAW sampled from Kumi in 2019 (Table 4).

#### 3.4.2. *Charops* cf. *Diversipes*

This species shared 99.8–100% nucleotide identity with *Charops* cf. *diversipe*s reported from Ghana (GenBank MN900729). It was recovered from FAW samples collected from Kasese and Bukedea. 

#### 3.4.3. *Sturmiopsis parasitica*

*Sturmiopsis parasitica* recovered from our study shared 99.35% identity with previously reported *S. parasitica* from Zimbabwe (GenBank DQ336399) (Table 1). This species was recovered in Oyam District in Northern Uganda in 2017 (Table 3) and from Sembabule District in Central Uganda in 2019 (Table 4).

#### 3.4.4. *Drino quadrizonula*

This tachinid shared 99.28–99.8% sequence identity with *Drino quadrizonula* (GenBank MN907776) (Table 1) from the Barcode of Life database [51]. This species was recovered from five districts in Central, Eastern, and Northern Uganda in 2017 (Table 3). In the 2019 survey, it was recovered from Kumi and Namutumba in Eastern Uganda (Table 4).

### 3.5. Relative Abundance and Parasitism of Spodoptera Frugiperda Eggs and Larvae

**Figure 3:**
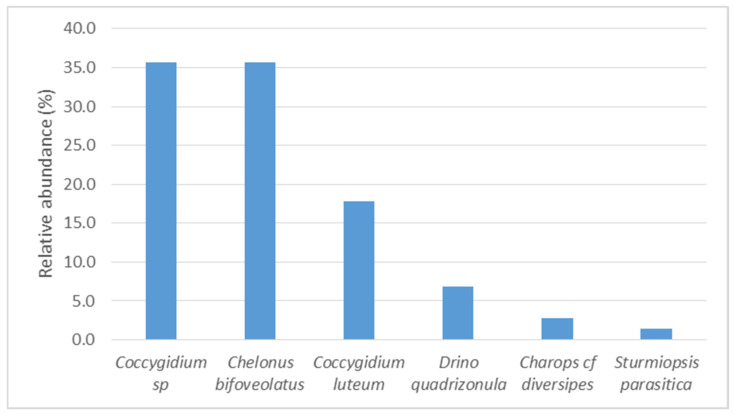
Relative abundance of parasitoid species of *Spodoptera frugiperda* larvae in 2017 across all surveyed districts in Uganda.

**Figure 4:**
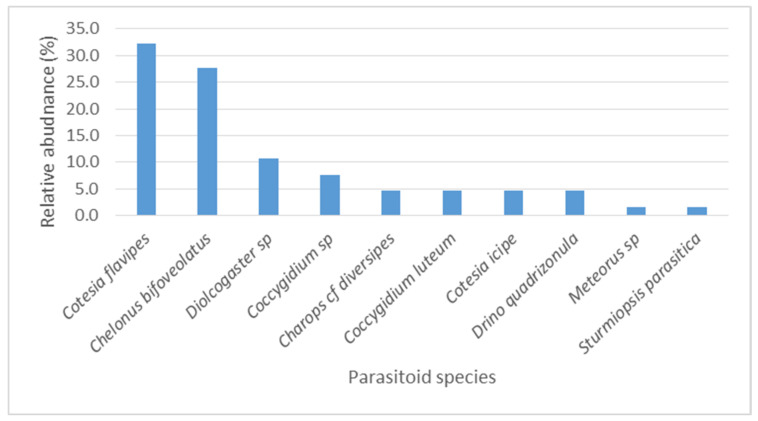
Relative abundance of parasitoid species of *Spodoptera frugiperda* larvae in 2019 across all surveyed districts in Uganda.

In the 2017, survey, the most abundant parasitoids of FAW were *Coccygidium* spp. and *Che. bifoveolatus* spp. (Figure 3). The other larval parasitoids were rare. In 2019, *Cot. flavipes* and *Che. bifoveolatus* were the most abundant (Figure 4). The other rare but recovered species in 2019 were *Coccygidium* spp., *Charops* cf. *diversipes*, *Cot. icipe*, *D. quadrizonula*, *Meteorus* sp., and *S. parasitica*.

Egg parasitism was not evaluated as counts were not made of the total number of eggs or neonates that emerged. Total larval parasitism ranged from 1.3% to 50% in 2017 (Table 3) and 0.8% to 33% in 2019 (Table 4). In 2017, total larval parasitism was highest in the central region (50%), and lower elsewhere: eastern (10.2%), northern (8.9%), and western (8.6%) regions. In 2019, total larval parasitism was also highest in the central region (19.2%), and lower in western (9.7%), northern (7.2%), and eastern (6.6%) regions.

## 4. Discussions

We recovered 13 species of parasitoids attacking FAW in different regions of Uganda. These belong to three families of Hymenoptera: Platygastridae (three species), Braconidae (seven species) and Ichneumonidae (one species) and one Dipteran family (two species of Tachinidae). The most widely distributed and abundant parasitoids in our study were *Coccygidium* and *Chelonus* spp. In this study, we also report (for the first time) parasitism by *S. parasitica*, *Diolcogaster* sp., *Drino quadrizonula*, and *Cot. flavipes* on FAW in maize crops in East Africa. Parasitism was averaged as 9.2% across all the locations and sampling times.

### 4.1. Coccygidium spp.

In our study, we report two *Coccygidium* species (*Coccygidium luteum* and an unknown putative novel *Coccygidium* sp. MT784160) parasitizing FAW in Uganda (Figure 2). *Coccygidium luteum* has previously been reported from Cameroon, Congo, Democratic Republic of Congo, Ethiopia, Ghana, Guinea, Kenya, Madagascar, Mauritius, Mozambique, Namibia, Niger, Nigeria, Rodriques Island, Réunion, Senegal, Seychelles, Somalia, South Africa, Tanzania, and Yemen in Asia [37,39,60,61]. This parasitoid reduced FAW leaf consumption by up to 89% under laboratory conditions and was thus identified as a candidate biological control agent for augmentative release [62]. There is, however, no report on the augmentative release of *Coccygidium* in Africa. In India, FAW is reported to be parasitized by *Coc. transcaspicum* in north-western India [63] and *Coc. melleum* in southern India [64] (both Hymenoptera: Braconidae).

In the review by Ranga-Rao et al. [65], *Coc. luteum*, *Coc. siss oo* (Hymenoptera: Braconidae) and *Coc. melleum* were reported to parasitize *Spodoptera exigua* larvae. Sequences from GenBank and iBoL revealed the occurrence of other *Coccygidium* spp. in other geographical regions (e.g., *Coc. phaeoscapos* (Hymenoptera: Braconidae) (Thailand); *Coccygidium* sp. (Townsville, Australia, HM433941), with at least 20 *Coccygidium* species known in Australia [66]. The existence of many species of *Coccygidium* distributed across the current invasive range of FAW suggests that some of the endemic *Coccygidium* species could potentially be candidate agents for control of FAW, although species recorded from South East Asia (e.g., Thailand) and Oceania (e.g., Australia) appeared basal to the African *Coccygidium* species that have been identified to parasitize FAW (see Figure 2). Despite the importance of *Coccygidium* parasitoids on economically important *Spodoptera* species, there remains a significant knowledge gap on species identity and the ecological services they provide for managing FAW in various parts of Africa, S.E. Asia, and Australia.

### 4.2. Chelonus Bifoveolatus

The *Chelonus* species recorded in Uganda matched the partial mtCOI sequences of *Che. bifoveolatus* that had previously been reported from FAW in Ghana, Benin, and Tanzania [39,67]. This widespread *Chelonus* species recovered in various regions of Uganda also matched partial mtCOI sequences of unidentified *Chelonus* spp. from Asia including India (e.g., *Che. formosanus* (Hymenoptera: Braconidae), [36]), French Polynesia and Pakistan. The *Chelonus* genus is the most common and widely distributed parasitoid of FAW in the Americas and Africa. In Colombia, *Che. inuslaris* parasitizes eggs of FAW, but is very susceptible to insecticides such as chlorpyriphos, methomyl, and cypermethrin but not to Bt toxin [68]. In the Caribbean Islands, *Che. antillarum* Marshall (Hymenoptera: Braconidae) was reported parasitizing FAW [26].

*Chelonus* species are globally distributed (e.g., See [65,69]). In Australia, at least 45 endemic species have been reported that includes *Che. scrobiculatus* (Szépligeti) and *Che. anomala* (both Hymenoptera: Braconidae) (reported from Queensland and Western Australia, as well as the Caribbean nation of Trinidad and Tobago; ALA accessed 23 October 2019) (see Kittel et al. [69] for review). The mtCO1 sequences of unidentified Australian *Chelonus* species (KJ472543, KJ438520, KJ472553) showed 84.77 to 88.44% pairwise nucleotide similarity with the Ugandan *Che*. *bifoveolatus* (GenBank MT776316) across 444 bp of partial mtCOI gene, suggesting that different species occur in the two countries. Diversity of the *Chelonus* parasitoids across the recent invasive range of the FAW should be investigated as this parasitoid genus could represent significant biological resources that have adaptive traits to their respective geographic range and could be used effectively to manage FAW and related *Spodoptera* species (e.g., *S. litura* (Lepidoptera: Noctuidae), [65] across different ecoclimatic zones.

### 4.3. Telenomus spp.

We recovered *T. remus* and an unidentified species of *Telenomus*. *T. remus* has been reported globally based on publicly available sequences (e.g., from GenBank and iBoL [51,70]). In Africa, it has been recorded in South Africa, Benin, Ivory Coast, Niger, Kenya, and now also in Uganda (this study) [38,39]. The presence of *T. remus* in Uganda offers promise for the control of FAW eggs. Indeed, *T. remus* was identified as an effective parasitoid of FAW eggs capable of causing 64% and 78% parasitism in the field and screenhouse in Niger [71], respectively, and is known to occur in several other African countries [38]. Because of its occurrence in Africa, emphases should be placed on conducting more distribution, effectiveness, and mass production studies, rather than exploring options for importation into Africa.

The sequences of the second *Telenomus* sp. shared 95.92% sequence identity with our *T. remus* sequence, suggesting that this is likely a different *Telenomus* sp. whose sequence has not been reported. The identity of the unknown *Telenomus* sp. in our study remains to be ascertained. Nevertheless, this *Telenomus* species could be an endemic African/Ugandan *Telenomus* species that could also serve as important biological agents against the invasive FAW. Therefore, its biology, ecology, effectiveness, and amenability for mass production and augmentative release should also be studied for inclusion in FAW IPM.

### 4.4. Tachinidae spp.

We recovered two species of Tachinidae flies: *S. parasitica* and *D. quadrizonula*. This is the first report of *S. parasitica* parasitizing FAW. Earlier, *S. parasitica* was reported to parasitize *B. fusca* in Congo [72] and, *Eldana saccharina* Walker (Lepidoptera: Pyralidae), and *Busseola fusca* (Fuller) and *Sesamia* spp. (both Lepidoptera: Noctuidae) in Ghana [73]. In our study, only two individuals were recovered, thus making it a minor contributor to the population dynamics of FAW from our initial assessments. The second tachinid had a close match with *Drino quadrizonula* reported from Ghana and Benin [39]. The was also a minor parasitoid of FAW in Uganda and various countries it has been recovered from.

### 4.5. Cotesia spp.

Three Ugandan *Cotesia* matched the recently named *Cot. icipe*, which was reported as the most prevalent parasitoid of FAW in Ethiopia and was also recovered in Kenya [39]. One Ugandan sample also matched *Cot. flavipes* [74]. Originating from the Indo-Asia region, *Cot. flavipes* is economically important and has been introduced into Africa and the New World for biocontrol of stem borers [75]. Elsewhere, *Cot. Marginiventris* was recovered from *S. frugiperda* in Mexico [76], while *Cot. scotti* was reported as attacking *Spodoptera cosmioides* (Walker) (Lepidoptera: Noctuidae) and *Spodoptera eridania* (Stoll) (Lepidoptera: Noctuidae) in Brazil [77]. In sub-Saharan Africa, *Cot. icipe* has been shown in laboratory experiments to be effective against *Spodoptera* species although efficacies against *S. frugiperda* and *Spodoptera exempta* (Walker) (Lepidoptera: Noctuidae) remain to be assessed [42]. Despite the ecological importance of these braconid wasps, little is known of how they interact and respond to their lepidopteran hosts [78], their population size, population dynamics, and interactions with their natural enemies [79], factors that may underpin our understanding of how the parasitoid populations persist across the landscape to impact on their respective lepidopteran hosts.

### 4.6. Charops cf. Diversipes

*Charops ater* has been reported to widely parasitize FAW larvae in Tanzania and Kenya [37] in 2017. Our study identified the *Charops* species from FAW larval samples collected from Busia, Bukedea, Sironko, and Dokolo Districts as *C.* cf. *diversipes,* sharing 99.29–100% nucleotide identity with samples from Benin and Ghana [39]. This species of parasitoid could be further exploited for FAW control given its presence in several African countries and regions of Uganda.

### 4.7. Meteorus sp.

Only one individual of *Meteorus* sp. was recovered from FAW in Uganda. This suggests that it is a minor biocontrol agent of FAW in Uganda. *Meteorus* species have, however, been recovered from FAW on maize in Mexico [27,76], Florida (USA) [33], and Northern Sinaloa (Mexico) [80]. In most of these cases, it was a minor parasitoid. In Africa, *Meteorus* spp. have also been recovered from millet head miner moth *Heliocheilus albipunctella* De Joannis (Lepidoptera: Noctuidae)*,* a noctuid pest of pearl millet [57].

### 4.8. Diolcogaster sp.

This is the first record of *Diolcogaster* attacking FAW in Africa. These are largely undescribed species of Microgastrinae, with nine species described in the Nearctic realm [81] and two from the Americas [82]. This species is likely a minor parasitoid of FAW given a single recovery.

### 4.9. Parasitism of FAW Larvae

Our study has shown that indigenous parasitoids, including parasitoids of lepidopteran stemborers, were capable of parasitizing FAW in Uganda. This is promising and further suggests that biological control will have a vital role in the integrated management of FAW. The overall level of parasitism by the different parasitoids was generally low, averaging 9.2%. This compares with the 9.5% parasitism of FAW reported in Mozambique [83]. The parasitism rate by *Che. bifoveolatus* observed in this study (0.8% to 16.7%) was also generally lower than those reported in Ghana where parasitism ranged from 0% to 35.6% [39]. In Senegal, 10.9% parasitism of FAW larvae by *Chelonus* sp. was reported [84]. In the case of *Coc. luteum*, parasitism levels of 4.6, 5.0, and 8.3% were recorded in Ethiopia, Tanzania, and Kenya, respectively [37]. In Uganda, however, parasitism by *Coc. luteum* ranged from 3.3% to 13.6%. Parasitism by *Cot. icipe* ranged from 2.1% to 3.2% in our study, sharply contrasting with earlier studies that showed higher parasitism levels of 5.6% in Kenya and 22.8% to 45.3% in Ethiopia [37].

Many factors influence parasitism rates. In Uganda, farmers adopted heavy application of mainly organophosphate broad-spectrum insecticides to control FAW. This would likely impact the abundance of natural enemies of FAW. Indeed, parasitism was highest in untreated plots than in plots treated with biopesticides (*Metarhizium anisopliae* and *Beauveria bassiana*) and the synthetic insecticide flubendiamide in Tanzania [67]. Consequently, any attempt to integrate biologically based measures to manage FAW will require options that do not significantly impact the activity of parasitoids.

## 5. Study Limitations

Our study was designed to provide preliminary knowledge of FAW parasitoid species diversity in Uganda such that follow-up studies with meaningful and testable hypotheses could be undertaken. Nevertheless, while limited in scope and survey frequencies, it represents one of the first surveys in Uganda to understand the diversity of parasitoids with the capacity to use FAW as an alternative host. Our study highlights the need for more widespread and frequent surveys, and more detailed population dynamics studies not only for Africa but for the current geographic range of the invasive populations of FAW (e.g., S.E. Asia, Australia), as well as potential future spread range (e.g., South Pacific, southern Europe, the Near East, northern Africa). More surveys will aid in documenting additional natural enemies and their impact, while population dynamics studies are needed to understand the role of different factors on the incidence and abundance of FAW and its natural enemies. The study of egg parasitoids was not detailed in the present study, and focus should therefore be placed on identifying egg parasitoids and to assess their efficiency in the management of FAW. Where promising parasitoids are identified, it would be important to develop protocols for mass production and distribution to other parts of the country.

### Conclusions and Recommendations

We have shown that FAW is attacked by at least 13 species of parasitoids in the surveyed districts of Uganda. The most abundant parasitoids (i.e., *Coccygidium* and *Chelonus* spp.) and their use as candidate biocontrol agents for augmentative control and IPM programs and impact by synthetic insecticides and biopesticides remain areas to be explored. With the endemic and introduced parasitoid species capable of attacking FAW, the country should focus on understanding the tritrophic interactions between parasitoids, their FAW hosts and plant hosts, and ecological and biological factors, to harness the benefits afforded by the existing and most promising parasitoids in Uganda. There will be a need for more detailed studies to document indigenous parasitoid recruitment by FAW and to assess, under controlled conditions, the effectiveness of selected species for biological control.

## Figures and Tables

**Table 1:** Parasitoid species recovered from eggs and larvae of *Spodoptera frugiperda* collected from maize fields in Uganda, 2017 and 2019 and their sequence identity as compared with publicly available sequences from GenBank and iBoL entries.

Order, Family, and Species	GenBank Accession No. of Ugandan Parasitoids	Host Stage Attacked	Species with Closest NucleoTide Sequence Match	Percent Identity, and Reference GenBank Accession Number and iBoL Entries
**Hymenoptera: Platygastridae**				
*Telenomus remus* Dixon	MT780201, MT780202	Eggs	*Telenomus remus*	100% (MK533751) [38]
*Telenomus* sp.	MT782153 and MT782154	Eggs	*Telenomus remus*	96.6% (MK533751) [38]
*Platygastridae* sp.	MT784162	Eggs	*Telenomus* sp.	87.4% (KR878931)
**Hymenoptera: Braconidae**				
*Chelonus bifoveolatus*	MT776316 to MT776320	Egg/larvae	*Chelonus bifoveolatus*	99.8–100% (KX051941) [56]; MN900744, MN900743 [39])
*Coccygidium luteum*	MT784186, MT784187	Larvae	*Coccygidium. luteum*	99.64%; (MN900741) [39])
*Coccygidium* sp.	MT784160	Larvae	*Coccygidium* sp. 3	98.2%; (MF098368)
*Cotesia flavipes*	MT780220	Larvae	*Cotesia flavipes*	100% (JF865973) and 99.6% (MN900741; MN900728) [39]
*Cotesia icipe*	MT780217 to MT780219	Larvae	*Cotesia icipe*	100% (iBoL ID JMIC0355) and 99.6% (MN900741; MN900728) [39] and 100% (JMIC0355)
*Diolcogaster* sp.	MT784194	Larvae	*Diolcogaster* sp.	98.38% (HM397609)
*Meteorus* sp.	MT784161	Larvae/pupae	*Meteorus* sp.	99.84% (MF673599) [57])
**Hymenoptera: Ichneumonidae**				
*Charops* cf. *diversipes*	MT784179 to MT784183	Larvae	*Charops* cf. *diversipes*	99.8–100% (MN900729) [39]
**Diptera: Tachinidae**				
*Sturmiopsis parasitica*(Curran)	MT784184 and MT784185	Larvae/pupae	*Sturmiopsis* *parasitica*	99.35% (DQ336399)
*Drino quadrizonula*	MT784175 to MT784178	Larvae/pupae	*Drino quadrizonula*	99.28–99.8% (MN907776) [39])

**Table 2:** Occurrence and relative abundance of egg parasitoids recovered from *Spodoptera frugiperda* in different districts of Uganda in 2019. † Nucleotide identity to *Telenomus remus* (MK533751) at 96.6%.

Region	District	*Species*	Relative Abundance (%) (*n* = 35)
Eastern	Bukedea	*Telenomus remus*	28.6
	Soroti	*Telenomus* sp. †	14.3
Western	Ntoroko	Platygastridae	2.9
	Ntoroko	*Telenomus remus*	31.4
	Mbarara	*Telenomus* sp. †	22.9

**Table 3:** Total larvae and parasitism of *Spodoptera frugiperda* larvae collected from different districts of Uganda in 2017. No. par = total parasitoids recovered; Para = % parasitism; Chb = *Chelonus bifoveolatus*; Col = *Coccygidium luteum*; Cos = *Coccygidium* sp; Chd = *Charops* cf. *diversipes*; Spa = *Sturmiopsis parasitica*; Dri = *Drino quadrizonula.*

				Host Stage Attacked, Parasitoid Species					
				Egg/larva	Larva			Larva/pupa	
Region/District	No. Larvae Collected	No. Parasitoids Recovered	Overall Parasitism, %	Chb	Col	Cos	Cha	Spa	Dri
**Central**									
Sembabule	2	1	50						50
**Eastern**									
Amuria	33	1	3			3			
Bukedea	67	1	1.5			1.5			
Kaberamaido	29	3	10.3	6.9		3.4			
Kamuli	15	1	6.7			6.7			
Katakwi	35	4	11.4	8.6		2.9			
Kumi	42	5	11.9	4.8		4.8			2.4
Mayuge	46	2	4.3	4.3					
Namutumba	22	4	18.1		13.6				4.5
Pallisa	58	1	1.7		1.7				
Serere	32	1	3.1	3.1					
Sironko	40	1	2.5				2.5		
Soroti	30	3	10	3.3		6.7			
**Northern**									
Adjumani	14	2	14.3			14.3			
Alebtong	10	2	20	20					
Dokolo	32	2	6.3	3.1			3.1		
Gulu	24	1	4.2			4.2			
Kaabong	37	3	8.1	5.4		2.7			
Kole	30	1	3.3	3.3					
Kotido	42	4	9.5	9.5					
Lira	40	4	10		10				
Maracha	75	1	1.3			1.3			
Moroto	9	1	11.1						11.1
Moyo	23	3	13		13				
Nebbi	36	5	13.9			13.9			
Nwoya	26	2	7.6	3.8		3.8			
Oyam	19	2	10.6					5.3	5.3
Pader	35	4	11.4			11.4			
Pakwach	24	1	4.2	4.2					
Yumbe	39	2	5.1			5.1			
Zombo	36	2	5.6		5.6				
**Western**									
Kasese	35	3	8.6	8.6					

**Table 4:** Total larvae and parasitism of *Spodoptera frugiperda* larvae collected from different districts of Uganda in 2019. No. par = total parasitoids recovered; Para = parasitism; Chb = *Chelonus bifoveolatus*; Col = *Coccygidium luteum*; Cos = *Coccygidium* sp.; Cof = *Cotesia flavipes*; Coi = *Cotesia icipe*; Dio = *Diolcogaster* sp.; Met = *Meteorus* sp.; Chd = *Charops* cf. *diversipes*; Spa = *Sturmiopsis parasitica*; Dri = *Drino quadrizonula*.

				Host Stage Attacked, Parasitoid Species									
				Egg/Larva	Larva						Larva/Pupa		
Region/District	No. Larvae Collected	No. Parasitoids Recovered	Overall Parasitism, %	Chb	Col	Cos	Cof	Coi	Dio	Cha	Met	Spa	Dri
**Central**													
Mubende	20	1	5			5							
Sembabule	3	1	33.3									33.3	
**Eastern**													
Bukedea	21	1	4.8							4.8			
Busia	32	2	6.2					3.1		3.1			
Kaberamaido	24	1	4.2			4.2							
Kamuli	48	2	4.1			2.1		2.1					
Katakwi	19	1	5.3	5.3									
Kumi	27	3	11.1								3.7		7.4
Luuka	74	1	1.4	1.4									
Mayuge	49	1	2	2									
Namutumba	30	3	10		6.7								3.3
Serere	28	1	3.6	3.6									
Soroti	30	6	20	16.7	3.3								
**Northern**													
Alebtong	119	1	0.8	0.8									
Gulu	58	1	1.7			1.7							
Kole	59	10	17	5.1					11.9				
Nwoya	42	4	9.5	4.8		2.4	2.4						
**Western**													
Kasese	31	5	16.1	9.7				3.2		3.2			

## Data Availability

The data presented in this study are available on request from the corresponding author.

## Supplementary Materials

The following are available online at https://www.mdpi.com/2075-4450/12/2/121/s1, Figure S1. Platygastridae unrooted phylogeny as inferred from 560bp partial mtCOI sequences using IQ-Tree with 1,000 bootstrap replications to estimate branch node confidence. *Telenomus remus* sequences are (GenBank accession numbers: MT780201 and MT780202), and the unknown Ugandan *Telenomus* species is represented by (GenBank accession numbers: MT780153 and MT780154). The unknown Platygastridae from Uganda is represented by (GenBank accession number MT784162) and indicated by the red colored branch, with poor phylogeny placement together with the Indonesian Mymaridae (MH926817). Note that this is an unrooted tree (i.e., no outgroup was defined and included for analysis) and is meant to assist with estimating confidences of clustered (i. e., related) sequences within the large assemblage of parasitoids species within the *Telenomus* genera.

## References

[1] Goergen G., Kumar P.L., Sankung S.B., Togola A., Tamò M. (2016). First report of outbreaks of the fall armyworm *Spodoptera frugiperda* (J E Smith) (Lepidoptera: Noctuidae), a new alien invasive pest in West and Central Africa. PLoS ONE.

[2] Cock M.J.W., Beseh P.K., Buddie A.G., Cafá G., Crozier J. (2017). Molecular methods to detect *Spodoptera frugiperda* in Ghana, and implications for monitoring the spread of invasive species in developing countries. Sci. Rep..

[3] Food and Agriculture Organization (2018). Briefing Note on FAO Actions on Fall Armyworm.

[4] Nagoshi R.N., Koffi D., Agboka K., Tounou K.A., Banerjee R., Jurat-Fuentes J.L., Meagher R.L. (2017). Comparative molecular analyses of invasive fall armyworm in Togo reveal strong similarities to populations from the eastern United States and the Greater Antilles. PLoS ONE.

[5] Otim M.H., Tay W.T., Walsh T.K., Kanyesigye D., Adumo S., Abongosi J., Ochen S., Sserumaga J., Alibu S., Abalo G. (2018). Detection of sister-species in invasive populations of the fall armyworm *Spodoptera frugiperda* (Lepidoptera: Noctuidae) from Uganda. PLoS ONE.

[6] Lee G., Seo B.Y., Lee J., Kim H., Song J.H., Lee W. (2020). First Report of the fall armyworm, *Spodoptera frugiperda* (Smith, 1797) (Lepidoptera: Noctuidae), a new migratory pest in Korea. Korean J. Appl. Entomol..

[7] Uzayisenga B., Waweru B., Kajuga J., Karangwa P., Uwumukiza B., Edgington S., Thompson E., Offord L., Cafá G., Buddie A. (2018). First record of the fall armyworm, *Spodoptera frugiperda* (J.E. Smith, 1797) (Lepidoptera: Noctuidae), in Rwanda. Afr. Entomol..

[8] Day R., Abrahams P., Bateman M., Beale T., Clottey V., Cock M., Colmenarez Y., Corniani N., Early R., Godwin J. (2017). Fall armyworm: Impacts and implications for Africa. Outlooks Pest Manag..

[9] Chimweta M., Nyakudya I.W., Jimu L., Mashingaidze A.B. (2020). Fall armyworm [*Spodoptera frugiperda* (J.E. Smith)] damage in maize: Management options for flood-recession cropping smallholder farmers. Int. J. Pest Manag..

[10] Tambo J.A., Day R.K., Lamontagne-Godwin J., Silvestri S., Beseh P.K., Oppong-Mensah B., Phiri N.A., Matimelo M. (2020). Tackling fall armyworm (*Spodoptera frugiperda*) outbreak in Africa: An analysis of farmers’ control actions. Int. J. Pest Manag..

[11] Kumela T., Simiyu J., Sisay B., Likhayo P., Mendesil E., Gohole L., Tefera T. (2019). Farmers’ knowledge, perceptions, and management practices of the new invasive pest, fall armyworm (*Spodoptera frugiperda*) in Ethiopia and Kenya. Int. J. Pest Manag..

[12] Midega C.A.O., Pittchar J.O., Pickett J.A., Hailu G.W., Khan Z.R. (2018). A climate-adapted push-pull system effectively controls fall armyworm, *Spodoptera frugiperda* (J E Smith), in maize in East Africa. Crop Prot..

[13] Johnson S.J. (1987). Migration and the life history strategy of the fall armyworm, *Spodoptera frugiperda* in the western hemisphere. Int. J. Trop. Insect Sci..

[14] Bateman M.L., Day R.K., Luke B., Edgington S., Kuhlmann U., Cock M.J.W. (2018). Assessment of potential biopesticide options for managing fall armyworm (*Spodoptera frugiperda*) in Africa. J. Appl. Entomol..

[15] Yu S.J. (1991). Insecticide resistance in the fall armyworm, *Spodoptera frugiperda* (J. E. Smith). Pestic. Biochem. Physiol..

[16] Carvalho R.A., Omoto C., Field L.M., Williamson M.S., Bass C. (2013). Investigating the molecular mechanisms of organophosphate and pyrethroid resistance in the fall armyworm *Spodoptera frugiperda*. PLoS ONE.

[17] Gutirrez-Moreno R., Mota-Sanchez D., Blanco C.A., Whalon M.E., Terán-Santofimio H., Rodriguez-Maciel J.C., Difonzo C. (2019). Field-evolved resistance of the fall armyworm (Lepidoptera: Noctuidae) to synthetic insecticides in Puerto Rico and Mexico. J. Econ. Entomol..

[18] Boaventura D., Martin M., Pozzebon A., Mota-Sanchez D., Nauen R. (2020). Monitoring of target-site mutations conferring insecticide resistance in *Spodoptera frugiperda*. Insects.

[19] Guan F., Zhang J., Shen H., Wang X., Padovan A., Walsh T.K., Tay W.T., Gordon K.H.J., James W., Czepak C. (2020). Whole-genome sequencing to detect mutations associated with resistance to insecticides and Bt proteins in *Spodoptera frugiperda*. Insect Sci..

[20] Zhang L., Liu B., Zheng W., Liu C., Zhang D., Zhao S., Li Z., Xu P., Wilson K., Withers A. (2020). Genetic structure and insecticide resistance characteristics of fall armyworm populations invading China. Mol. Ecol. Resour..

[21] Hruska A.J. (2019). Fall armyworm (*Spodoptera frugiperda*) management by smallholders. CAB Rev. Perspect. Agric. Vet. Sci. Nutr. Nat. Resour..

[22] Molina-Ochoa J., Lezama-Gutierrez R., Gonzalez-Ramirez M., Lopez-Edwards M., Rodriguez-Vega M.A., Arceo-Palacios F. (2003). Pathogens and parasitic nematodes associated with populations of fall armyworm (Lepidoptera: Noctuidae) larvae in Mexico. Fla. Entomol..

[23] Ruiz-Nájera R.E., Ruiz-Estudillo R.A., Sánchez-Yáñez J.M., Molina-Ochoa J., Skoda S.R., Coutiño-Ruiz R., Pinto-Ruiz R., Guevara-Hernández F., Foster J.E. (2013). Occurrence of entomopathogenic fungi and parasitic nematodes on *Spodoptera frugiperda* (Lepidoptera: Noctuidae) larvae collected in Central Chiapas, México. Fla. Entomol..

[24] Gardner W.A., Fuxa J.R. (1980). Pathogens for the suppression of the fall armyworm. Fla. Entomol..

[25] Camargo A.M., Castañera P., Farinós G.P., Huang F. (2017). Comparative analysis of the genetic basis of Cry1F resistance in two strains of *Spodoptera frugiperda* originated from Puerto Rico and Florida. J. Invertebr. Pathol..

[26] Ashley T.R. (1986). Geographical distribution and parasitization levels for parasitoids of the fall armyworm, *Spodoptera frugiperda*. Fla. Entomol..

[27] Molina-Ochoa J., Hamm J.J., Lezama-Gutiérrez R., López-Edwards M., González-Ramírez M., Pescador-Rubio A. (2001). A survey of fall armyworm (Lepidoptera: Noctuidae) parasitoids in the Mexican states of Michoacán, Colima, Jalisco, and Tamaulipas. Fla. Entomol..

[28] Wyckhuys K.A.G., O’Neil R.J. (2006). Population dynamics of *Spodoptera frugiperda* Smith (Lepidoptera: Noctuidae) and associated arthropod natural enemies in Honduran subsistence maize. Crop Prot..

[29] Murúa M.G., Molina-Ochoa J., Fidalgo P. (2009). Natural distribution of parasitoids of larvae of the fall armyworm, *Spodoptera frugiperda*, in Argentina. J. Insect Sci..

[30] Gutiérrez-Martínez A., Tolon-Becerra A., Lastra-Bravo X.B. (2012). Biological control of *Spodoptera frugiperda* eggs using *Telenomus remus* Nixon in maize-bean-squash polyculture. Am. J. Agric. Biol. Sci..

[31] Hardke J.T., Lorenz G.M., Leonard B.R. (2015). Fall armyworm (Lepidoptera: Noctuidae) ecology in Southeastern cotton. J. Integr. Pest Manag..

[32] Hay-Roe M.M., Meagher R.L., Nagoshi R.N., Newman Y. (2016). Distributional patterns of fall armyworm parasitoids in a corn field and a pasture field in Florida. Biol. Control.

[33] Meagher R.L., Nuessly G.S., Nagoshi R.N., Hay-Roe M.M. (2016). Parasitoids attacking fall armyworm (Lepidoptera: Noctuidae) in sweet corn habitats. Biol. Control.

[34] Liao Y.L., Yang B., Xu M.F., Lin W., Wang D.S., Chen K.W., Chen H.Y. (2019). First report of *Telenomus remus* parasitizing *Spodoptera frugiperda* and its field parasitism in southern China. J. Hymenopt. Res..

[35] Firake D.M., Behere G.T. (2020). Natural mortality of invasive fall armyworm, *Spodoptera frugiperda* (J. E. Smith) (Lepidoptera: Noctuidae) in maize agroecosystems of northeast India. Biol. Control.

[36] Firake D.M., Behere G.T. (2020). Bioecological attributes and physiological indices of invasive fall armyworm, *Spodoptera frugiperda* (J. E. Smith) infesting ginger (*Zingiber officinale* Roscoe) plants in India. Crop Prot..

[37] Sisay B., Simiyu J., Malusi P., Likhayo P., Mendesil E., Elibariki N., Wakgari M., Ayalew G., Tefera T. (2018). First report of the fall armyworm, *Spodoptera frugiperda* (Lepidoptera: Noctuidae), natural enemies from Africa. J. Appl. Entomol..

[38] Kenis M., du Plessis H., Van den Berg J., Ba M.N., Goergen G., Kwadjo K.E., Baoua I., Tefera T., Buddie A., Cafà G. (2019). *Telenomus remus*, a candidate parasitoid for the biological control of *Spodoptera frugiperda* in Africa, is already present on the continent. Insects.

[39] Agboyi L.K., Goergen G., Beseh P., Mensah S.A., Clottey V.A., Glikpo R., Buddie A., Cafà G., Offord L., Day R. (2020). Parasitoid complex of fall armyworm, *Spodoptera frugiperda*, in Ghana and Benin. Insects.

[40] Polaszek A., Kimani S.W. (1990). Telenomus species (Hymenopetra: Scelionidae) attacking eggs of pyralid pests (Lepidoptera) in Africa: A review and guide to identification. Bull. Entomol. Res..

[41] Shaw M.R., Huddleston T., Dolling W.R., Askew R. (1991). Classification and Biology of Braconid Wasps (Hymenoptera: Braconidae).

[42] Fiaboe K.K.M., Fernández-Triana J., Nyamu F.W., Agbodzavu K.M. (2017). *Cotesia icipe* Sp. N., a new Microgastrinae wasp (Hymenoptera: Braconidae) of importance in the biological control of Lepidopteran pests in Africa. J. Hymenopt. Res..

[43] Aguirre H., de Almeida L.F., Shaw S.R., Sarmiento C.E. (2015). An illustrated key to Neotropical species of the genus *Meteorus* Haliday (Hymenoptera, Braconidae, Euphorinae). Zookeys.

[44] Gauld I.D. (1984). An introduction to the Ichneumonidae of Australia.

[45] O’Hara J.E., Shima H., Zhang C. (2009). Annotated catalogue of the Tachinidae (Insecta: Diptera) of China. Zootaxa.

[46] O’Hara J.E., Cerretti P. (2016). Annotated catalogue of the Tachinidae (Insecta, Diptera) of the Afrotropical Region, with the description of seven new genera. Zookeys.

[47] Yao H., Song J., Liu C., Luo K., Han J., Li Y., Pang X., Xu H., Zhu Y., Xiao P. (2010). Use of ITS2 region as the universal DNA barcode for plants and animals. PLoS ONE.

[48] Staden R., Beal K.F., Bonfield J.K. (2000). The Staden package, 1998. Methods Mol. Biol..

[49] Kearse M., Moir R., Wilson A., Stones-Havas S., Cheung M., Sturrock S., Buxton S., Cooper A., Markowitz S., Duran C. (2012). Geneious Basic: An integrated and extendable desktop software platform for the organization and analysis of sequence data. Bioinformatics.

[50] Sayers E.W., Beck J., Brister J.R., Bolton E.E., Canese K., Comeau D.C., Funk K., Ketter A., Kim S., Kimchi A. (2020). Database resources of the National Center for Biotechnology Information. Nucleic Acids Res..

[51] Ratnasingham S., Hebert P.D.N. (2007). BOLD: The Barcode of Life Data System: Barcoding. Mol. Ecol. Notes.

[52] Katoh K., Misawa K., Kuma K.I., Miyata T. (2002). MAFFT: A novel method for rapid multiple sequence alignment based on fast Fourier transform. Nucleic Acids Res..

[53] Katoh K., Standley D.M. (2013). MAFFT multiple sequence alignment software version 7: Improvements in performance and usability. Mol. Biol. Evol..

[54] Trifinopoulos J., Nguyen L.T., von Haeseler A., Minh B.Q. (2016). W-IQ-TREE: A fast online phylogenetic tool for maximum likelihood analysis. Nucleic Acids Res..

[55] Huson D.H., Scornavacca C. (2012). Dendroscope 3: An interactive tool for rooted phylogenetic trees and networks. Syst. Biol..

[56] Ramage T., Martins-Simoes P., Mialdea G., Allemand R., Duplouy A., Rousse P., Davies N., Roderick G.K., Charlat S. (2017). A DNA barcode-based survey of terrestrial arthropods in the Society Islands of French Polynesia: Host diversity within the SymbioCode project. Eur. J. Taxon..

[57] Sow A., Brévault T., Delvare G., Haran J., Benoit L., d’Acier A.C., Galan M., Thiaw C., Soti V., Sembène M. (2018). DNA sequencing to help identify crop pests and their natural enemies in agro-ecosystems: The case of the millet head miner *Heliocheilus albipunctella* (Lepidoptera: Noctuidae) in sub-Saharan Africa. Biol. Control.

[58] Sharkey M.J., Chapman E.G. Phylogeny of the Agathidinae (Hymenoptera: Braconidae) with a revised tribal classification and the description of a new genus. Proceedings of the Entomological Society of Washington.

[59] Sharkey M.J., Chapman E.G. (2015). The Nearctic genera of Agathidinae (Hymenoptera: Braconidae) with a phylogenetic analysis, illustrated generic key, and the description of three new genera. Zootaxa.

[60] Koffi D., Kyerematen R., Eziah V.Y., Agboka K., Adom M., Goergen G., Meagher R.L. (2020). Natural enemies of the fall armyworm, *Spodoptera frugiperda* (J.E. Smith) (Lepidoptera: Noctuidae) in Ghana. Fla. Entomol..

[61] Van Noort S. WaspWeb: Hymenoptera of the Afrotropical Region. Iziko Museums of South Africa. www.waspweb.org.

[62] Agboyi L.K., Mensah S.A., Clottey V.A., Beseh P., Glikpo R., Rwomushana I., Day R., Kenis M. (2019). Evidence of leaf consumption rate decrease in fall armyworm, *Spodoptera frugiperda*, larvae parasitized by *Coccygidium luteum*. Insects.

[63] Gupta A., Soujanya P.L., van Achterberg C., Sekhar J.C. (2020). *Coccygidium transcaspicum* (Kokujev) (Hymenoptera: Braconidae) parasitizing larvae of invasive pest *Spodoptera frugiperda* (J. E. Smith) (Lepidoptera: Noctuidae) in India. Zootaxa.

[64] Sharanabasappa D., Kalleshwaraswamy C.M., Poorani J., Maruthi M.S., Pavithra H.B., Diraviam J. (2019). Natural enemies of *Spodoptera frugiperda* (J. E. Smith) (Lepidoptera: Noctuidae), a recent invasive pest on maize in South India. Fla. Entomol..

[65] Rao G.V.R., Wightman J.A., Rao D.V.R. (1993). World review of the natural enemies and diseases of *Spodoptera litura* (F.) (Lepidoptera: Noctuidae). Int. J. Trop. Insect Sci..

[66] Stevens N.B., Austin A.D., Jennings J.T. (2010). Synopsis of Australian Agathidine wasps (Hymenoptera: Braconidae: Agathidinae). Zootaxa.

[67] Ngangambe M.H., Mwatawala M.W. (2020). Effects of entomopathogenic fungi (EPFs) and cropping systems on parasitoids of fall armyworm (*Spodoptera frugiperda*) on maize in eastern central, Tanzania. Biocontrol Sci. Technol..

[68] Zenner I., Álvarez A., Barreto S. (2006). Influence of parasitism by *Chelonus insularis* Cresson (Hymenoptera: Braconidae) on the susceptibility of *Spodoptera frugiperda* (J.E. Smith) (Lepidoptera: Noctuidae) to insecticides. Neotrop. Entomol..

[69] Kittel R.N., Austin A.D., Klopfstein S. (2016). Molecular and morphological phylogenetics of Chelonine parasitoid wasps (Hymenoptera: Braconidae), with a critical assessment of divergence time estimations. Mol. Phylogenet. Evol..

[70] Zhang Z., Schwartz S., Wagner L., Miller W. (2000). A greedy algorithm for aligning DNA sequences. J. Comput. Biol..

[71] Laminou S.A., Ba M.N., Karimoune L., Doumma A., Muniappan R. (2020). Parasitism of locally recruited egg parasitoids of the fall armyworm in Africa. Insects.

[72] Kankonda O.M., Akaibe B.D., Sylvain N.M., Le Ru B.P. (2018). Response of maize stemborers and associated parasitoids to the spread of grasses in the rainforest zone of Kisangani, DR Congo: Effect on stemborers biological control. Agric. Entomol..

[73] Hordzi W.H.K. (2018). Lepidopterous stem borers of maize (Zea mays): Agro-ecological and regional composition and distribution of their parasitoids in Southern Ghana. J. Biosci. Biotechnol. Discov..

[74] Smith M.A., Fernández-Triana J.L., Eveleigh E., Gómez J., Guclu C., Hallwachs W., Hebert P.D.N., Hrcek J., Huber J.T., Janzen D. (2013). DNA barcoding and the taxonomy of Microgastrinae wasps (Hymenoptera, Braconidae): Impacts after 8 years and nearly 20 000 sequences. Mol. Ecol. Resour..

[75] Muirhead K.A., Murphy N.P., Sallam N., Donnellan S.C., Austin A.D. (2012). Phylogenetics and genetic diversity of the *Cotesia flavipes* complex of parasitoid wasps (Hymenoptera: Braconidae), biological control agents of Lepidopteran stemborers. Mol. Phylogenet. Evol..

[76] Jourdie V., Alvarez N., Turlings T.C.J. (2008). Identification of seven species of Hymenopteran parasitoids of *Spodoptera frugiperda*, using polymerase chain reaction amplification and restriction enzyme digestion. Agric. Entomol..

[77] De Freitas J.G., Takahashi T.A., Figueiredo L.L., Fernandes P.M., Camargo L.F., Watanabe I.M., Foerster L.A., Fernandez-Triana J., Shimbori E.M. (2019). First record of *Cotesia scotti* (Valerio and Whitfield, 2009) (Hymenoptera: Braconidae: Microgastrinae) comb. nov. parasitising *Spodoptera cosmioides* (Walk, 1858) and *Spodoptera eridania* (Stoll, 1782) (Lepidoptera: Noctuidae) in Brazil. Rev. Bras. Entomol..

[78] Desneux N., Ramírez-Romero R., Bokonon-Ganta A.H., Bernal J.S. (2010). Attraction of the parasitoid *Cotesia marginiventris* to host (*Spodoptera frugiperda*) frass is affected by transgenic maize. Ecotoxicology.

[79] Van Nouhuys S., Tay W.T. (2001). Causes and consequences of small population size for a specialist parasitoid wasp. Oecologia.

[80] López M.A., Martínez-Castillo A.M., García-Gutiérrez C., Cortez-Mondaca E., Escobedo-Bonilla C.M. (2018). Parasitoids and entomopathogens associated with fall armyworm, *Spodoptera frugiperda*, in Northern Sinaloa. Southwest. Entomol..

[81] Yu D.S.K., van Achterberg C., Horstmann K. (2016). Taxapad 2016, Ichneumonoidea 2015.

[82] Fernandez-Triana J. (2018). Ten unique and charismatic new species of microgastrinae wasps (Hymenoptera: Braconidae) from North America. Zookeys.

[83] Caniço A., Mexia A., Santos L. (2020). First report of native parasitoids of fall armyworm *Spodoptera frugiperda* smith (Lepidoptera: Noctuidae) in Mozambique. Insects.

[84] Tendeng E., Labou B., Diatte M., Djiba S., Diarra K. (2019). The fall armyworm *Spodoptera frugiperda* (J.E. Smith), a new pest of maize in Africa: Biology and first native natural enemies detected. Int. J. Biol. Chem. Sci..

