# Supplementary material for: Parasitoid Distribution and Parasitism of the Fall Armyworm Spodoptera frugiperda (Lepidoptera: Noctuidae) in Different Maize Producing Regions of Uganda
Source: Insects. 2021 Jan 29;12(2):121. doi: 10.3390/insects12020121 (PMC7912086; doi:10.3390/insects12020121)

Figure S1. Platygastridae unrooted phylogeny as inferred from 560bp partial mtCOI sequences using IQ-Tree with 1,000 bootstrap replications to estimate branch node confidence. *Telenomus remus* sequences are (GenBank accession numbers: MT780201 and MT780202), and the unknown Ugandan *Telenomus* species is represented by (GenBank accession numbers: MT780153 and MT780154). The unknown Platygastridae from Uganda is represented by (GenBank accession number MT784162) and indicated by the red coloured branch, with poor phylogeny placement together with the Indonesian Mymaridae (MH926817). Note that this is an unrooted tree (i.e., no outgroup was defined and included for analysis) and is meant to assist with estimating confidences of clustered (i. e., related) sequences within the large assemblage of parasitoids species within the *Telenomus* genera.

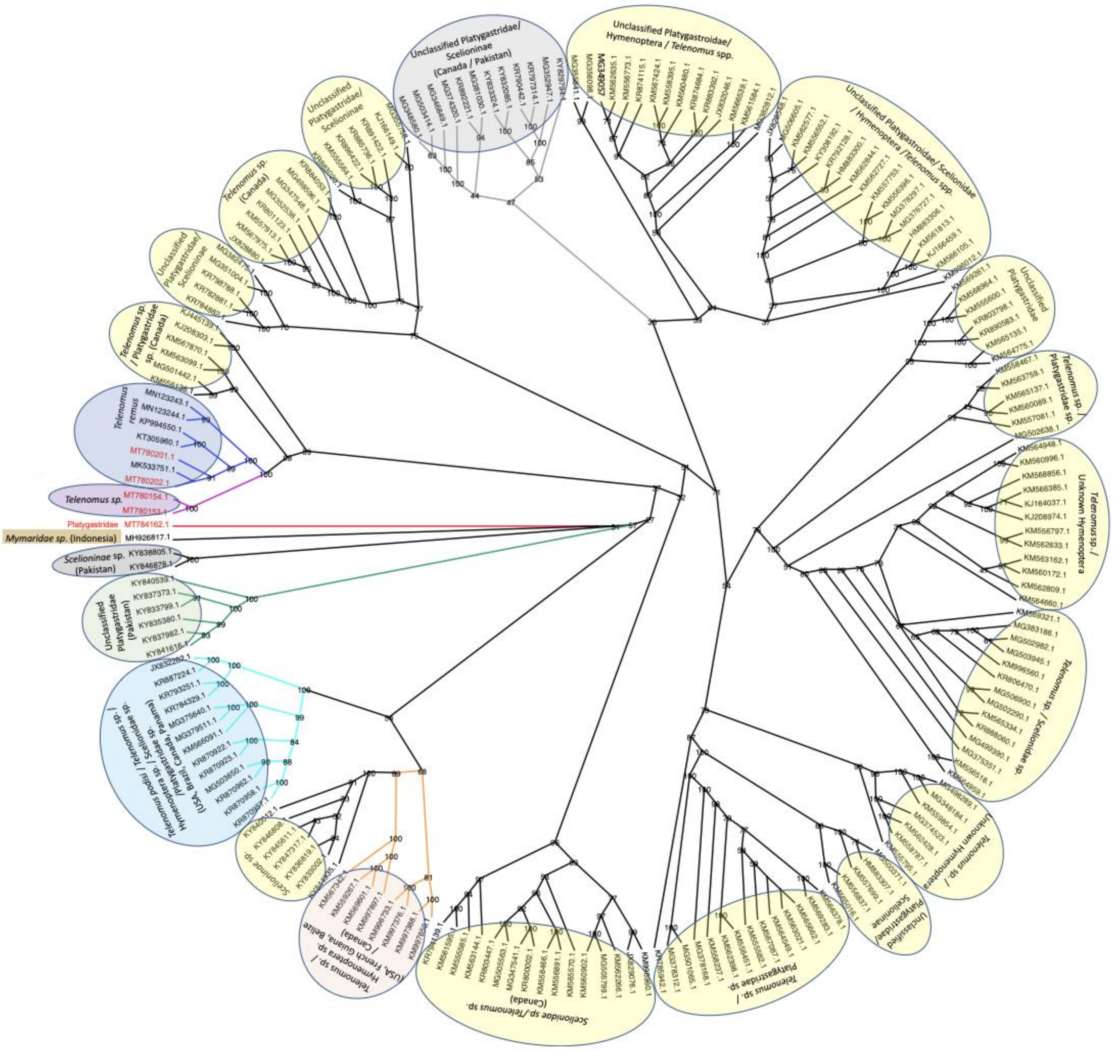

Supplement: Supplementary file 1 [file insects-12-00121-s001.pdf]
